# Supplementary material for: A Herpesvirus of Turkey-Based Vector Vaccine Reduces Transmission of Newcastle Disease Virus in Commercial Broiler Chickens with Maternally Derived Antibodies
Source: Vaccines (Basel). 2020 Oct 16;8(4):614. doi: 10.3390/vaccines8040614 (PMC7720113; doi:10.3390/vaccines8040614)
Supplement: Supplementary file 1 [file vaccines-08-00614-s001.zip › File S1- R-code supplement.zip!/NDV_supplement.html]

NDV\_supplement with `A vector vaccine reduces transmission of Newcastle disease virus in commercial broiler chickens with maternally derived antibodies’


# NDV\_supplement with `A vector vaccine reduces transmission of Newcastle disease virus in commercial broiler chickens with maternally derived antibodies’

#### T. Tatar-Kis, E.A.J. Fischer, C. Cazaban, E. Kovacs, Z. G. Homonnay, V. Palya and J.A.(Arjan) Stegeman

#### 10-7-2020

## Used libraries and functions

#libraries

```
options("getSymbols.warning4.0"=FALSE)
#add libraries
library(openxlsx)
```

```
## Warning: package 'openxlsx' was built under R version 3.6.2
```

```
library(ggplot2)
```

```
## Warning: package 'ggplot2' was built under R version 3.6.2
```

```
library(lme4)
```

```
## Loading required package: Matrix
```

```
## Warning: package 'Matrix' was built under R version 3.6.2
```

```
library(survival)
```

```
## Warning: package 'survival' was built under R version 3.6.2
```

# User defined functions

```
#colour-blind colour palette
cbPalette <- c("#999999", "#E69F00", "#56B4E9", "#009E73", "#F0E442", "#0072B2", "#D55E00", "#CC79A7")

#function to reform data for glm
source("NDV_datamodel.R")
```

## Load and transform data

```
#set directory with data equal to the working directory
data.dir <- getwd()
#read the data of the vaccination group

#data original CT values
ndvdata <- read.xlsx("Appendix_individualsCtvaluesandevaluation.xlsx", 
                 startRow =2,
                 colNames = T)

#cevadata.original <- read.xlsx(paste0(data.dir, "Appendix-individuals Ct values and evaluation.xlsx"),
 #                       sheet = "RAW (4)")
ndvdata<-fill.type.na.rename(ndvdata)
#data used for transmission 
#cevadata <- read.xlsx(paste0(data.dir, "DataNDVtrial_manual_Egil 20200317_Timea20200319.xlsx"),
 #                       sheet = "Input_seedernotchanged")

#transform to usable data
data.one.SIR <- reform.SIR.data(data = ndvdata,
                                sampleday.vars= paste0(c(1:14),".dpch"),
                                exclude.seeder = T )
data.one.SIR$Vaccinated <- factor(data.one.SIR$Vaccinated, levels = c("Yes","No"))
data.one.SIR$Vaccinated<-relevel(data.one.SIR$Vaccinated, ref = "No")
```

## Excretion

For those that were defined positive

```
ct.data <- ndvdata
for(day in c(6:19)){
  
  ct.data[(!data.one.SIR[[2]][,day]==1)|is.na(data.one.SIR[[2]][,day]),day]<- NA
  
}

suppressWarnings(
 ct.data$AUC <- sapply(c(1:length(ct.data$Group)), function(j){sum(36-as.numeric(ct.data[j,6:19]), na.rm = T)}) )#do not bother about NA's

#selection only the ct values >0
ct.data<- ct.data[ct.data$AUC>0,]
 
ctauc <- aggregate(formula = AUC ~ Vaccinated+ci+Sample,
          data = ct.data[ct.data$AUC>0,],
          FUN = "mean", rm.na = T)
ctauc$sd<-aggregate(formula = AUC ~ Vaccinated+ci+Sample,
          data = ct.data[ct.data$AUC>0,],
          FUN = "sd")$AUC
ctauc$n<-aggregate(formula = AUC ~ Vaccinated+ci+Sample,
          data = ct.data[ct.data$AUC>0,],
          FUN = "length")$AUC
#combine all categories
ct.data$category <- paste0(ct.data$Vaccinated,".",ct.data$ci)

#overall test for AUC against either one of the categories (categories is combination type bird and vaccination status)
kruskal.test(formula = AUC ~ category,data = ct.data[ct.data$Sample == "ON swab",])
```

```
## 
##  Kruskal-Wallis rank sum test
## 
## data:  AUC by category
## Kruskal-Wallis chi-squared = 26.077, df = 3, p-value = 9.189e-06
```

```
kruskal.test(formula = AUC ~ category,data = ct.data[ct.data$Sample == "CLO swab",])
```

```
## 
##  Kruskal-Wallis rank sum test
## 
## data:  AUC by category
## Kruskal-Wallis chi-squared = 22.131, df = 3, p-value = 6.127e-05
```

Compare challenge type (seeder or contact) stratisfied by type of swab.

```
#cloacal swabs contact versus seeders
wilcox.test(formula = AUC ~ Challenge,data = ct.data[ct.data$Sample == "CLO swab",], paired = F)
```

```
## 
##  Wilcoxon rank sum test
## 
## data:  AUC by Challenge
## W = 36, p-value = 3.895e-05
## alternative hypothesis: true location shift is not equal to 0
```

```
#oro-nasal swabs contact versus seeders
wilcox.test(formula = AUC ~ Challenge,data = ct.data[ct.data$Sample == "ON swab",], paired = F)
```

```
## 
##  Wilcoxon rank sum test
## 
## data:  AUC by Challenge
## W = 147, p-value = 0.01814
## alternative hypothesis: true location shift is not equal to 0
```

Compare challenge type (seeder or contact) stratisfied by type of swab in unvaccinated birds.

```
#test  seed=contact CLO in unvaccinated
wilcox.test(formula = AUC ~ Challenge,data = ct.data[ct.data$Sample == "CLO swab"&ct.data$Vaccinated =="No",], paired = F)
```

```
## 
##  Wilcoxon rank sum test
## 
## data:  AUC by Challenge
## W = 21, p-value = 0.0001785
## alternative hypothesis: true location shift is not equal to 0
```

```
#test  seed=contact ON in unvaccinated
wilcox.test(formula = AUC ~ Challenge,data = ct.data[ct.data$Sample == "ON swab"&ct.data$Vaccinated =="No",], paired = F)
```

```
## 
##  Wilcoxon rank sum test
## 
## data:  AUC by Challenge
## W = 39, p-value = 0.006081
## alternative hypothesis: true location shift is not equal to 0
```

Compare challenge type (seeder or contact) stratisfied by type of swab in vaccinated birds.

```
#test seed - contact per treatment CLO in vaccinated
 wilcox.test(formula = AUC ~ Challenge,data = ct.data[ct.data$Sample == "CLO swab"&ct.data$Vaccinated =="Yes",], paired = F)
```

```
## 
##  Wilcoxon rank sum test
## 
## data:  AUC by Challenge
## W = 1, p-value = 0.1905
## alternative hypothesis: true location shift is not equal to 0
```

```
 ct.data[ct.data$Sample == "CLO swab"&ct.data$Vaccinated =="No"&ct.data$ci=="seeder",]$AUC
```

```
##  [1] 49.36 52.36 52.45 49.40 41.10 26.98 33.86 37.55 17.89 37.49 44.51 39.97
## [13] 54.71 26.33 43.02
```

```
 ct.data[ct.data$Sample == "CLO swab"&ct.data$Vaccinated =="No"&ct.data$ci=="contact",]$AUC
```

```
##  [1] 18.06 24.20 19.36  3.23 12.48 17.24  2.48 19.45 28.13 43.63  8.31 24.32
## [13] 34.52
```

```
 #test seed - contact per treatment ON in vaccinated
 wilcox.test(formula = AUC ~ Challenge,data = ct.data[ct.data$Sample == "ON swab"&ct.data$Vaccinated =="Yes",], paired = F)
```

```
## 
##  Wilcoxon rank sum test
## 
## data:  AUC by Challenge
## W = 9, p-value = 0.009667
## alternative hypothesis: true location shift is not equal to 0
```

```
  ct.data[ct.data$Sample == "ON swab"&ct.data$Vaccinated =="No"&ct.data$ci=="seeder",]$AUC
```

```
##  [1] 54.49 57.83 57.13 55.31 63.68 40.68 17.92 50.90 30.07 13.18 45.42 66.35
## [13] 32.33 52.23 41.89
```

```
 ct.data[ct.data$Sample == "ON swab"&ct.data$Vaccinated =="No"&ct.data$ci=="contact",]$AUC
```

```
##  [1] 28.91 13.77 11.01  5.13 46.40 19.85  0.33 30.98 49.20 42.20 30.17 37.97
## [13] 35.33
```

Compare vaccinated to unvaccinated stratisfied by type of swab.

```
#test vaccinated vs unvaccinated overall
wilcox.test(formula = AUC ~ Vaccinated,data = ct.data[ct.data$Sample == "CLO swab",])
```

```
## 
##  Wilcoxon rank sum test
## 
## data:  AUC by Vaccinated
## W = 180, p-value = 0.0002165
## alternative hypothesis: true location shift is not equal to 0
```

```
wilcox.test(formula = AUC ~ Vaccinated,data = ct.data[ct.data$Sample == "ON swab",])
```

```
## 
##  Wilcoxon rank sum test
## 
## data:  AUC by Vaccinated
## W = 426, p-value = 1.934e-06
## alternative hypothesis: true location shift is not equal to 0
```

Compare vaccinated to unvaccinated stratisfied by type of swab for contact birds .

```
#test vaccinated versus unvaccinated for contact

wilcox.test(formula = AUC ~ Vaccinated,data = ct.data[ct.data$Sample == "CLO swab"&ct.data$ci =="contact",])
```

```
## 
##  Wilcoxon rank sum test
## 
## data:  AUC by Vaccinated
## W = 62, p-value = 0.001634
## alternative hypothesis: true location shift is not equal to 0
```

```
wilcox.test(formula = AUC ~ Vaccinated,data = ct.data[ct.data$Sample == "ON swab"&ct.data$ci =="contact",])
```

```
## 
##  Wilcoxon rank sum test
## 
## data:  AUC by Vaccinated
## W = 82, p-value = 0.002425
## alternative hypothesis: true location shift is not equal to 0
```

```
#test vaccinated versus unvaccinated for seeder
wilcox.test(formula = AUC ~ Vaccinated,data = ct.data[ct.data$Sample == "CLO swab"&ct.data$ci =="seeder",])
```

```
## 
##  Wilcoxon rank sum test
## 
## data:  AUC by Vaccinated
## W = 29, p-value = 0.02941
## alternative hypothesis: true location shift is not equal to 0
```

```
wilcox.test(formula = AUC ~ Vaccinated,data = ct.data[ct.data$Sample == "ON swab"&ct.data$ci =="seeder",])
```

```
## 
##  Wilcoxon rank sum test
## 
## data:  AUC by Vaccinated
## W = 140, p-value = 8.505e-05
## alternative hypothesis: true location shift is not equal to 0
```

## Infectious period

# Data selection

```
#get data to calculate infectious period
out.indiv.one.SIR<- data.one.SIR[[2]];
#sum all positive intervals
out.indiv.one.SIR$infper <- apply(data.one.SIR[[2]][,c(5:18)]==1, 1, sum, na.rm = T);
#remove 0 as these are not infected thus should not be considered
out.indiv.one.SIR[out.indiv.one.SIR$infper == 0,]$infper<- NA #zero means never been infected
```

# Plot

```
#make factor
out.indiv.one.SIR$Vaccinated<-factor(out.indiv.one.SIR$Vaccinated, levels = c("Yes","No"))

hist.one.chal <- ggplot(data = out.indiv.one.SIR) +
  geom_histogram(aes(x = infper, y = ..density.. , fill = Vaccinated), colour = "black",
                 position = "dodge",binwidth = 1, na.rm = T)+
  scale_fill_manual(values=c("Yes" = cbPalette[2],"No" = cbPalette[3]))+
  scale_x_continuous(breaks = c(0:10))+
 # ggtitle("Distribution of infectious period")+
  ylab("Proportion of excreting chickens")+
  xlab("Infectious period (days)") +
  #theme(panel.background = NULL,  legend.position = "none")+
  theme_bw()+
  facet_grid( ~ Challenge,
             labeller = labeller(
       # Vaccinated = c("No" = "Unvaccinated", "Yes" = "Vaccinated"),
        Challenge = c("contact" = "Contact", "seeder" = "Seeder")
    )) 
ggsave("InfectiousPeriod.tiff",hist.one.chal, dpi =720)
```

```
## Saving 7 x 5 in image
```

```
hist.one.chal
```

# Estimate infectious period

The infectious period \(T\_{inf}\): Parametric survival analyses assuming Normal distribution.

```
#add censoring as variable to the data set
out.indiv.one.SIR$cens <- 1-as.numeric(out.indiv.one.SIR$`14.dpch` == 1)
out.indiv.one.SIR$cens[is.na(out.indiv.one.SIR$cens)] <- 1
out.indiv.one.SIR[out.indiv.one.SIR$cens==0,]
```

```
##     Vaccinated Group      ci bird.id   Sample 1.dpch 2.dpch 3.dpch 4.dpch
## 186        Yes    1A contact    7325 category      0      0      0      0
## 228        Yes    1B contact    7349 category      0      0      0      0
##     5.dpch 6.dpch 7.dpch 8.dpch 9.dpch 10.dpch 11.dpch 12.dpch 13.dpch 14.dpch
## 186      0      0      0      0      0       0       0       0       1       1
## 228      0      0      0      0      0       0       0       0       1       1
##         titer evaluation Challenge infper cens
## 186  43.37047   negative   contact      1    0
## 228 200.50405   negative   contact      1    0
```

```
#Survival analyses on contact birds
#perform exponential survival analysis
surv.fit.one.SIR.exponential <- survival::survreg(survival::Surv(infper,cens)~ relevel(Vaccinated, ref= "No"), dist = "exponential",data = out.indiv.one.SIR[out.indiv.one.SIR$Challenge == "contact",], na.action = na.omit)

#perform gaussian survival analysis 
surv.fit.one.SIR.gaussian <- survival::survreg(survival::Surv(infper,cens)~ relevel(Vaccinated, ref= "No") , dist = "gaussian",data = out.indiv.one.SIR[out.indiv.one.SIR$Challenge == "contact",], na.action = na.omit)
AIC(surv.fit.one.SIR.exponential)
```

```
## [1] 139.1386
```

```
AIC(surv.fit.one.SIR.gaussian)
```

```
## [1] 100.046
```

Based on AIC select gaussian model.

```
#check if dropping vaccination changes the model
drop1(surv.fit.one.SIR.gaussian,test = "Chisq")
```

```
## Single term deletions
## 
## Model:
## survival::Surv(infper, cens) ~ relevel(Vaccinated, ref = "No")
##                                 Df    AIC    LRT Pr(>Chi)  
## <none>                             100.05                  
## relevel(Vaccinated, ref = "No")  1 102.52 4.4776  0.03434 *
## ---
## Signif. codes:  0 '***' 0.001 '**' 0.01 '*' 0.05 '.' 0.1 ' ' 1
```

```
#print confidence intervals
summary(surv.fit.one.SIR.gaussian)
```

```
## 
## Call:
## survival::survreg(formula = survival::Surv(infper, cens) ~ relevel(Vaccinated, 
##     ref = "No"), data = out.indiv.one.SIR[out.indiv.one.SIR$Challenge == 
##     "contact", ], na.action = na.omit, dist = "gaussian")
##                                     Value Std. Error     z      p
## (Intercept)                         4.762      0.299 15.95 <2e-16
## relevel(Vaccinated, ref = "No")Yes -1.387      0.621 -2.23  0.025
## Log(scale)                          0.314      0.135  2.33  0.020
## 
## Scale= 1.37 
## 
## Gaussian distribution
## Loglik(model)= -47   Loglik(intercept only)= -49.3
##  Chisq= 4.48 on 1 degrees of freedom, p= 0.034 
## Number of Newton-Raphson Iterations: 4 
## n=29 (12 observations deleted due to missingness)
```

```
Tinfci<-confint(surv.fit.one.SIR.gaussian)
```

drop1(surv.fit.one.SIR.gaussian,test = “Chisq”) summary(surv.fit.one.SIR.gaussian.sc)

# Calculate beta and R0

```
#pre select data without rows for S =0 or I = 0 or S = NA or C = NA
out.nona.one.SIR <- data.one.SIR[[1]][(!is.na(data.one.SIR[[1]]$S)&!is.na(data.one.SIR[[1]]$C) &data.one.SIR[[1]]$I>0 &data.one.SIR[[1]]$S>0),]
out.nona.one.SIR$Vaccinated <- factor(out.nona.one.SIR$Vaccinated, levels = c("No","Yes"))
out.nona.one.SIR$Vaccinated <- relevel(out.nona.one.SIR$Vaccinated, ref = "No")
#estimate beta 
fit.SIR <- glm(cbind(C, S-C) ~ relevel(Vaccinated,ref = "No") ,
                offset = log(I/N),
                family = binomial(link = "cloglog"), data = out.nona.one.SIR, 
                na.action = na.omit)

#calculate R0
alpha = 0.05 #conf.level
#beta
mean.logbeta <- cumsum(summary(fit.SIR)$coefficients[,1])
var.logbeta <- summary(fit.SIR)$coefficients[,2]^2
confint.logbeta<-  confint(fit.SIR)+c(0,mean.logbeta[1])
```

```
## Waiting for profiling to be done...
```

```
#infectious period
mean.inf.per <- cumsum(summary(surv.fit.one.SIR.gaussian)$coefficients[1:2])
confint.inf.per <- confint(surv.fit.one.SIR.gaussian)[1:2,]+c(0, mean.inf.per[1])
#get the variance from the profile likelihood
var.inf.per <- ((confint.inf.per-mean.inf.per)[,2]/qnorm(0.05/2))^2
#calculate log of the mean and the variance of the log of the mean. 
mean.log.inf.per <- log(mean.inf.per)
var.log.inf.per <- log(1+var.inf.per/(mean.inf.per)^2)

#calculate R
mean.logR <- mean.logbeta + mean.log.inf.per
var.logR <- var.logbeta + var.log.inf.per
cll.logR <- mean.logR - qnorm(1-0.05/2) * sqrt(var.logR)
cul.logR <- mean.logR + qnorm(1-0.05/2) * sqrt(var.logR)

#put an a table
data.table <- data.frame(Tinf = round(mean.inf.per,2),
          Tinfll = round(confint.inf.per[,1],2),
          Tinful = round(confint.inf.per[,2],2),
          beta = round(exp(mean.logbeta),2),
          betall = round(exp(confint.logbeta)[,1],2),
          betaul =round(exp(confint.logbeta)[,2],2),
          R0 = round(exp(mean.logR),2),
          R0ll = round(exp (cll.logR),2),
          R0ul = round(exp(cul.logR),2))
row.names(data.table)<- c("Unvaccinated","Vaccinated")
out.table <- knitr::kable(x = data.table,
             digits =2,
             format = "html",
             #row.names = c("Unvaccinated","Vaccinated")),
             col.names =c("Estimate","2.5%","97.5%",
                          "Estimate","2.5%","97.5%",
                          "Estimate","2.5%","97.5%"))
kableExtra::add_header_above(out.table,c("Treatment" =1,
                 "Tinf" = 3,
                 "beta" = 3,
                 "R0" = 3))
```

| Treatment | Tinf | | | beta | | | R0 | | |
| --- | --- | --- | --- | --- | --- | --- | --- | --- | --- |
|  | Estimate | 2.5% | 97.5% | Estimate | 2.5% | 97.5% | Estimate | 2.5% | 97.5% |
| Unvaccinated | 4.76 | 4.18 | 5.35 | 0.67 | 0.42 | 1.01 | 3.20 | 2.06 | 4.96 |
| Vaccinated | 3.38 | 2.16 | 4.59 | 0.27 | 0.13 | 0.52 | 0.91 | 0.42 | 1.94 |

Probability of a major outbreak is 0 for \(R\_0<= 1\) and $1-1/R\_0 $ otherwise. This is an approximation, because the infectious period is normally distributed.

```
#approximate probability major outbreak
(1-1/data.table$R0 ) #negative means 0
```

```
## [1]  0.6875000 -0.0989011
```

```
(1-1/data.table$R0ll ) #negative means 0
```

```
## [1]  0.5145631 -1.3809524
```

```
(1-1/data.table$R0ul ) #negative means 0
```

```
## [1] 0.7983871 0.4845361
```

The final size can still be calculated as the absolute number of infections by one initiall infected \(\dfrac{1}{1-R\_0}\) or the fraction of the population \(z\) eventually infected \(-\ln(1-z) = z R\_0\) :

```
#approximate final size of the outbreak
final.size<- function(R0){if(R0<1){return(1/(1-R0))}else{
 return(uniroot(function(z){-R0*z-log(1-z)},lower = .1, upper =1)$root)
  }
}
sapply(data.table$R0,final.size) #first is fraction, second is number
```

```
## [1]  0.9525546 11.1111111
```

```
sapply(data.table$R0ll,final.size) #first is fraction, second is number
```

```
## [1] 0.8124057 1.7241379
```

```
sapply(data.table$R0ul,final.size) #both fractions
```

```
## [1] 0.9927278 0.7796435
```
